# Clinical Study on the Treatment of Somatisation Disorder With Repetitive Transcranial Magnetic Stimulation Combined With Venlafaxine

**DOI:** 10.62641/aep.v53i6.2041

**Published:** 2025-12-17

**Authors:** Shaolan Wan, Jiang Li, Yan Liao, Liang Wen

**Affiliations:** ^1^Department of Clinical Psychology, The Third People’s Hospital of Yichun, 336000 Yichun, Jiangxi, China

**Keywords:** transcranial magnetic stimulation, venlafaxine, somatisation disorder, combined therapy, clinical efficacy, safety

## Abstract

**Background:**

Somatisation disorder (SD) is a chronic and complex mental health condition characterized by persistent somatic symptoms lacking a clear organic basis, frequently co-occurring with anxiety and depressive disorders. Venlafaxine, a serotonin-norepinephrine reuptake inhibitor, is a standard pharmacotherapy, but its efficacy as monotherapy can be limited. Repetitive transcranial magnetic stimulation (rTMS) is a non-invasive neuromodulation technique that has shown promise for various neuropsychiatric conditions. However, evidence regarding the combined application of rTMS and venlafaxine for SD remains scarce. This study aimed to evaluate the efficacy and safety of rTMS combined with venlafaxine in the treatment of SD.

**Methods:**

This retrospective study analysed clinical data from 126 patients admitted with SD to the Third People’s Hospital of Yichun from September 2022 to November 2023. Patients were classified into two groups according to the treatment regimens administered during hospitalization, rather than pre-specified study grouping: a treatment group (n = 63) that received venlafaxine in conjunction with rTMS, and a control group (n = 63) that received venlafaxine monotherapy. Clinical outcomes were evaluated using the Hamilton Anxiety Scale (HAMA), Symptom Checklist 90 (SCL-90), Clinical Global Impression–Severity of Illness Scale (CGI-SI) and Hamilton Depression Scale (HAMD) at baseline (T0) and at weeks 1 (T1), 2 (T2), 4 (T3) and 6 (T4) after treatment initiation. Adverse events were documented and analysed. The study analysed factors affecting treatment efficacy through univariate and multivariate logistic regression analyses.

**Results:**

The treatment group exhibited a statistically significant improvement in overall therapeutic efficacy relative to the control group (*p* < 0.05). Both groups demonstrated significant decreases in HAMD, HAMA, SCL-90 and CGI-SI scores at all post-treatment time points (T1, T2, T3 and T4) compared with baseline (T0). At each follow-up time point, the treatment group exhibited significantly lower scores on all assessment scales relative to the control group (*p* < 0.05). Both groups experienced adverse reactions, but the treatment group exhibited a lower incidence of these events (*p* < 0.05). Univariate analysis revealed that patients in the ineffective group were more likely to have received venlafaxine monotherapy, to be older and to have lower baseline HAMA (T0) scores than the effective group (all *p* values < 0.05). Multivariate logistic regression revealed that venlafaxine monotherapy (odds ratio [OR] = 3.181, 95% confidence interval [CI] [1.184–8.549]) and baseline HAMA score (OR = 0.784, 95% CI [0.644–0.954]) are significant factors affecting clinical efficacy (*p* < 0.05).

**Conclusions:**

The combination of rTMS and venlafaxine demonstrates superior efficacy and an improved safety profile compared to venlafaxine monotherapy in treating SD, indicating its potential for wider clinical use. Clinicians should monitor patients’ psychological status to reduce adverse reactions and improve treatment adherence.

## Introduction

Somatisation disorder (SD), or the Briquet syndrome, is a multifaceted chronic mental disorder resulting from various contributing factors. This condition is marked by recurring and diverse pain and somatic discomfort without organic pathological basis. It is frequently associated with notable anxiety and depression [1, 2]. Patients often seek medical consultation for ongoing somatic symptoms and anxiety. Despite normal examination results or reassurance from the physician regarding the absence of organic disease, doubts persist. The severity of the symptoms reported frequently exceeds clinical assessment, seriously affecting daily functioning. In severe cases, individuals may experience a loss of typical social functioning [2]. The main treatment for these patients typically involves a combination of pharmacotherapy and psychotherapy [3]. Venlafaxine, which inhibits the reuptake of norepinephrine (NE) and serotonin (5-HT), has demonstrated substantial antidepressant and anxiolytic properties and is widely recognized for its therapeutic benefits [4]. Some patients exhibit poor efficacy with a single drug, and adherence to treatment is challenging because of low compliance and evident side effects. Therefore, synergistic approaches are frequently employed in clinical practice to enhance therapeutic efficacy.

Repetitive transcranial magnetic stimulation (rTMS) is a non-invasive neurostimulation technique that has received considerable attention for its application in the treatment of mental and neurological disorders. no instances of its use in the treatment of SD has been documented. rTMS influences the cerebral cortex via pulsed magnetic fields, inducing changes in neuronal electrical activity and exerting excitatory or inhibitory effects. It bidirectionally modulates brain function, has a high safety profile and can partially compensate for the limitations of drug therapy [5, 6]. Different frequencies in rTMS produce varying therapeutic effects. High-frequency rTMS (≥5 Hz) and low-frequency rTMS (≤1 Hz) represent two distinct modalities of transcranial magnetic stimulation. High-frequency rTMS enhances cortical excitability, whereas low-frequency rTMS reduces neuronal activity, allowing for the bidirectional modulation of brain excitation and inhibition [7]. Theoretically, the combination of rTMS and venlafaxine for the treatment of SD may use their individual benefits and address the limitations of monotherapy, potentially improving therapeutic outcomes. Currently, research on this combined treatment regimen is lacking. Hence, this study aims to examine the feasibility, effectiveness and safety of combining rTMS with venlafaxine for treating SD treatment and establish a scientific basis for its clinical application.

## Materials and Methods

### Materials

The clinical data of 126 patients admitted with SD in Third People’s Hospital from September 2022 to November 2023 were retrospectively selected.

The inclusion criteria were as follows: (1) meeting the diagnostic criteria for SD as outlined in the tenth edition of the *International Classification of Diseases* (ICD-10) [8]; (2) age of 18–65 years; (3) absence of organic brain disease or other significant somatic conditions; and (4) no dependence on psychotropic medications; The exclusion criteria were as follows: (1) severely poor physical health conditions; (2) cardiac pacemakers, hearing aids or cochlear implants; (3) history of epileptic seizures or syncope; (4) increased intracranial pressure or metallic objects in the skull; (5) pregnancy or lactation; (6) allergy to venlafaxine; and (7) uncontrolled hypertension defined as a systolic blood pressure of ≥140 mmHg, or a diastolic blood pressure of ≥90 mmHg and severe complications associated with hypertension, including renal insufficiency and heart failure. Patients were categorised according to the treatment regimens administered during hospitalisation rather than to the pre-defined groupings in the study. The treatment group (n = 63) received venlafaxine in conjunction with rTMS, and the control group (n = 63) received only venlafaxine. The ethics committee of the hospital approved this study. All patients or their legal guardians in cases of impaired decision-making capacity due to severe symptoms provided written informed consent prior to treatment. The consent form explicitly outlined the study’s purpose (assessing the efficacy and safety of venlafaxine in conjunction with rTMS), intervention details (venlafaxine dosage adjustment plan and rTMS parameter settings), potential risks (minor adverse reactions like nausea or dizziness) and data utilisation (de-identified for research purposes only). Participants were made aware of their right to withdraw from the study at any point without impacting their future clinical care, and all consent processes adhered to the Declaration of Helsinki.

### Treatment Methods

Control Group: Patients received treatment with venlafaxine tablets (Kanghong Pharmaceutical, National Drug Approval No. H20070269, specification: 75 mg per tablet). The initial dosage was 75 mg/day, which was gradually increased to 150 mg/day within the first week according to individual circumstances, and the treatment duration was a total of 6 weeks.

Treatment group: Based on the control group, the final dosages for the two groups were 136.4 ± 30.2 and 131.6 ± 29.8 mg/day, respectively. No significant difference was observed between the two groups (t = 0.898, *p* = 0.371). Patients in the intervention group received rTMS treatment, produced by Yirende Medical Equipment New Technology Co., Ltd., Wuhan, China, model: YRDCCY-1. A combination of high-frequency stimulation excitation and low-frequency stimulation inhibition was employed for patients with SD. Stimulation at a frequency of 15 Hz [9] was applied to the right prefrontal cortex using a figure-of-eight coil with a diameter of 5.5 cm, at 100% of the motor threshold. A total of 600 pulses were delivered in each train, comprising 30 pulses at 20 s intervals, culminating in a treatment duration of 10 minutes. The treatment was conducted five times weekly over a duration of 6 weeks.

### Assessment Indicators

Main Outcome Measures: The Symptom Checklist 90 (SCL-90) assesses the overall psychological and physical symptoms of patients [10]. Psychological status was assessed prior to treatment (T0) and at 1 (T1), 2 (T2), 4 (T3) and 6 weeks (T4) after treatment. The SCL-90 comprises 90 items scored 1–5 points, resulting in a maximum possible score of 450 points. A decrease in SCL-90 scores post-intervention indicates a reduction in clinical symptoms.

Secondary outcome indicators: (1) The psychological status of patients at T0 and T1, T2, T3, T4 was evaluated using the Hamilton Anxiety Scale (HAMA) [11] and Hamilton Depression Scale (HAMD) [12]. The HAMA contains 14 items scored 0–4 points, with a total score of 56 points. The HAMD uses the original 17 versions of the HAMA in 1960, with a total score of 0–52 points. Low HAMA and HAMD scores after intervention indicate improved the psychological state.

(2) Comparison of Disease Severity and Symptom Intensity: Both groups were evaluated with the Clinical Global Impression (CGI) scale [13] at T0, T1, T2, T3 and T4. The CGI scale consists of two components: Severity of Illness (SI) and Global Improvement (GI). The SI reflects the degree of symptom severity and is rated on a scale of 1–7. High scores indicate pronounced psychiatric symptoms. The GI reflects overall therapeutic change and is scored from 1 to 7. High scores denote deterioration in condition. This study used the Clinical Global Impression–Severity of Illness Scale (CGI-SI) score assessment. In addition to interviews, the patient’s past medical records were considered in the evaluation of the CGI-SI score.

(3) Safety Assessment: The incidence of adverse reactions in the two groups of patients after 6 weeks of treatment was counted. The Treatment Emergent Symptom Scale (TESS) [14] was used in the assessment of recorded adverse reactions after treatment at T1, T2, T3 and T4. These reactions included the incidence of nausea, somnolence and constipation. Severity was assessed using a five-point scale (0–4; high scores indicate severe adverse reactions).

(4) Evaluation of Clinical Efficacy After 6 Weeks of Treatment: The clinical outcomes for both groups were evaluated as follows: (i) Marked improvement was defined as the recovery of daily living abilities and the disappearance of cognitive dysfunction, and the SCL-90 score decreased by more than 50%; (ii) improvement was characterised by a notable reduction in clinical symptoms, corresponding to a decrease of 25%–50% in the SCL-90 score; (iii) no improvement was indicated by minimal changes in clinical symptoms, and a reduction of less than 25% was found in the SCL-90 score. The overall effectiveness rate was determined using the formula [(number of significant improvements + number of improvements)/total number of cases] × 100%. According to the above evaluation results, the patients were divided into ineffective and effective groups. The factors that may affect the clinical efficacy were preliminarily screened by univariate analysis, and then independent factors affecting the clinical efficacy were further explored using a multivariate logistic regression model.

### Statistical Analysis

Statistical analysis was conducted using SPSS Version 22.0 (IBM Corp., Armonk, NY, USA). All charts in the study, including efficacy score trend diagrams and adverse reaction incidence comparison tables, were generated using GraphPad Prism 8 (GraphPad Software, San Diego, CA, USA). Normality of all data was assessed using the Shapiro–Wilk method. Continuous variables exhibiting a normal distribution are expressed as mean ± standard deviation ($\bar{x}$ ± s) and analysed between groups using the independent-samples *t*-test. Continuous variables exhibiting a non-normal distribution are reported as median and interquartile range (M [Q1, Q3]), with comparisons between groups conducted using the Mann–Whitney U test. Categorical variables were summarised as percentages and analysed using chi-square (χ^2^) tests. On the basis of HAMD, HAMA, SCL-90 and CGI scores, temporal changes within each group (experimental and control) were compared using repeated-measures one-way analysis of variance (ANOVA) across different time points. Mauchly’s test was performed to validate the sphericity assumption required for repeated-measures ANOVA. In instances where this assumption was not satisfied (*p* < 0.05), the degrees of freedom were modified through the Greenhouse–Geisser correction. Potential influencing factors were evaluated through univariate analysis (*p* < 0.05) and subsequently analysed using multivariate logistic regression to determine independent predictors of therapeutic effectiveness. A *p* value threshold of <0.05 was established to indicate statistical significance.

## Results

### General Information Comparison 

No significant differences were observed between the two groups regarding gender, age, duration of illness, marital status, baseline SCL-90 somatisation score, history of previous psychotropic medication use, concurrent somatic diseases, education level and social support status (*p* > 0.05). This finding suggests that the two groups were comparable in terms of baseline characteristics (Table 1).

**Table 1.** **General information comparison**.

General information		Treatment group (n = 63)	Control group (n = 63)	t/χ^2^	*p*
Age ($\bar{x}$ ± s, years)		31.41 ± 9.62	32.18 ± 10.70	0.768	0.446
Gender (n [%])	Male	24 (38.10)	26 (41.27)	0.133	0.716
	Female	39 (61.90)	37 (58.73)		
Duration of Illness ($\bar{x}$ ± s, years)		4.52 ± 1.77	4.33 ± 1.22	0.694	0.489
Education Level (n [%])	Primary school or below	8 (12.70)	10 (15.87)	0.287	0.866
	Junior high to high school	32 (50.79)	30 (47.62)		
	College and above	23 (36.51)	23 (36.51)		
Marital status (n [%])	Married	12 (19.05)	21 (33.33)	0.698	0.705
	Unmarried	23 (36.51)	25 (39.68)		
	Divorced/Widowed	28 (44.44)	17 (26.98)		
History of Previous Psychotropic Medication (n [%])	Yes	15 (23.81)	17 (26.98)	0.168	0.682
	No	48 (76.19)	46 (73.02)		
Concurrent Somatic Disease (n [%])	Yes	9 (14.29)	11 (17.46)	0.238	0.626
	No	54 (85.71)	52 (82.54)		
Social Support Status (n [%])	Low level	8 (12.70)	9 (14.29)	0.069	0.966
	Medium level	48 (76.19)	47 (74.60)		
	High level	7 (11.11)	7 (11.11)		
Baseline SCL-90 Somatic Symptom Score		202.37 ± 24.05	199.38 ± 26.79	0.658	0.512
Baseline HAMA Score		27.00 (25.00, 29.00)	27.00 (24.00, 28.00)	2.479	0.115
Baseline HAMD Score		26.10 ± 2.48	25.54 ± 2.71	1.200	0.232
Baseline CGI-SI Score		6.00 (5.00, 6.00)	6.00 (5.00, 6.00)	0.412	0.521
Baseline TESS Score		7.00 (7.00, 9.00)	8.00 (7.00, 9.00)	1.703	0.192

Notes: SCL-90, Symptom Checklist 90; HAMA, Hamilton Anxiety Rating Scale; HAMD, Hamilton Depression Rating Scale; CGI-SI, Clinical Global Impression-Severity of Illness; TESS, Treatment Emergent Symptom Scale.

### Comparison of HAMD and HAMA Scores Before and After Treatment

**Fig. 1.**
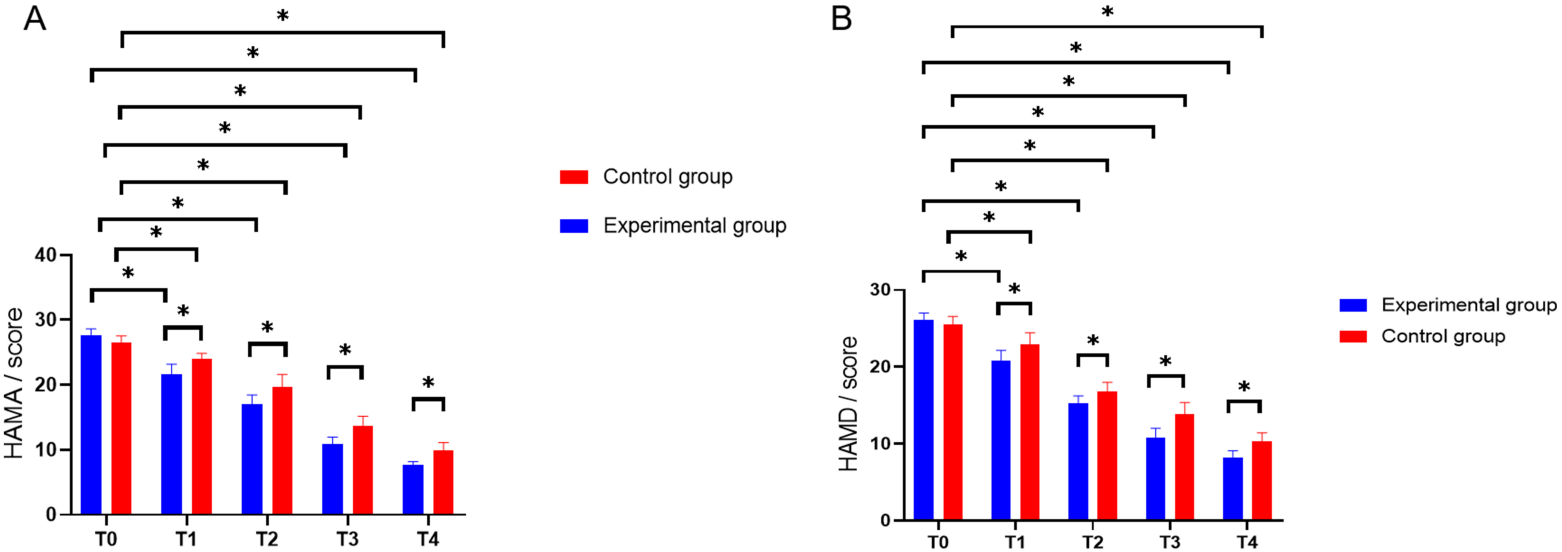
**Contrast between pre-intervention and post-intervention HAMD and HAMA scores**. Note: *p < 0.05 (after Bonferroni correction); (A) HAMA; (B) HAMD; HAMA, Hamilton Anxiety Rating Scale; HAMD, Hamilton Depression Rating Scale.

After treatment, both groups demonstrated a significant decrease in the HAMD and HAMA scale scores (*p* < 0.05). Furthermore, a comparison of the HAMD and HAMA scores between the two groups prior to treatment revealed no significant differences (*p* > 0.05). At T1, T2, T3 and T4, the treatment group exhibited significantly lower HAMD and HAMA scores compared to the control group (*p* < 0.01, after Bonferroni correction; Fig. 1). A repeated-measures ANOVA was performed to assess the differences in HAMD scores at various time points for both groups. Mauchly’s sphericity test indicated a violation of the sphericity assumption for HAMD and HAMA scores across different time points in the two groups (*p* < 0.05). The Greenhouse–Geisser correction results demonstrated that for HAMA scores, the main effects of group, measurement times and their interaction effects were all significant (F1 = 35.916, F2 = 657.277, F3 = 6.453, *p* < 0.05, after Bonferroni correction). In a similar manner, the analysis of HAMD scores across various time points in the two groups revealed significant main effects for both group and measurement times, as well as a significant interaction effect (F1 = 21.720, F2 = 579.522, F3 = 5.587, *p* < 0.05, after Bonferroni correction).

### Comparison of SCL-90 Scores

**Fig. 2.**
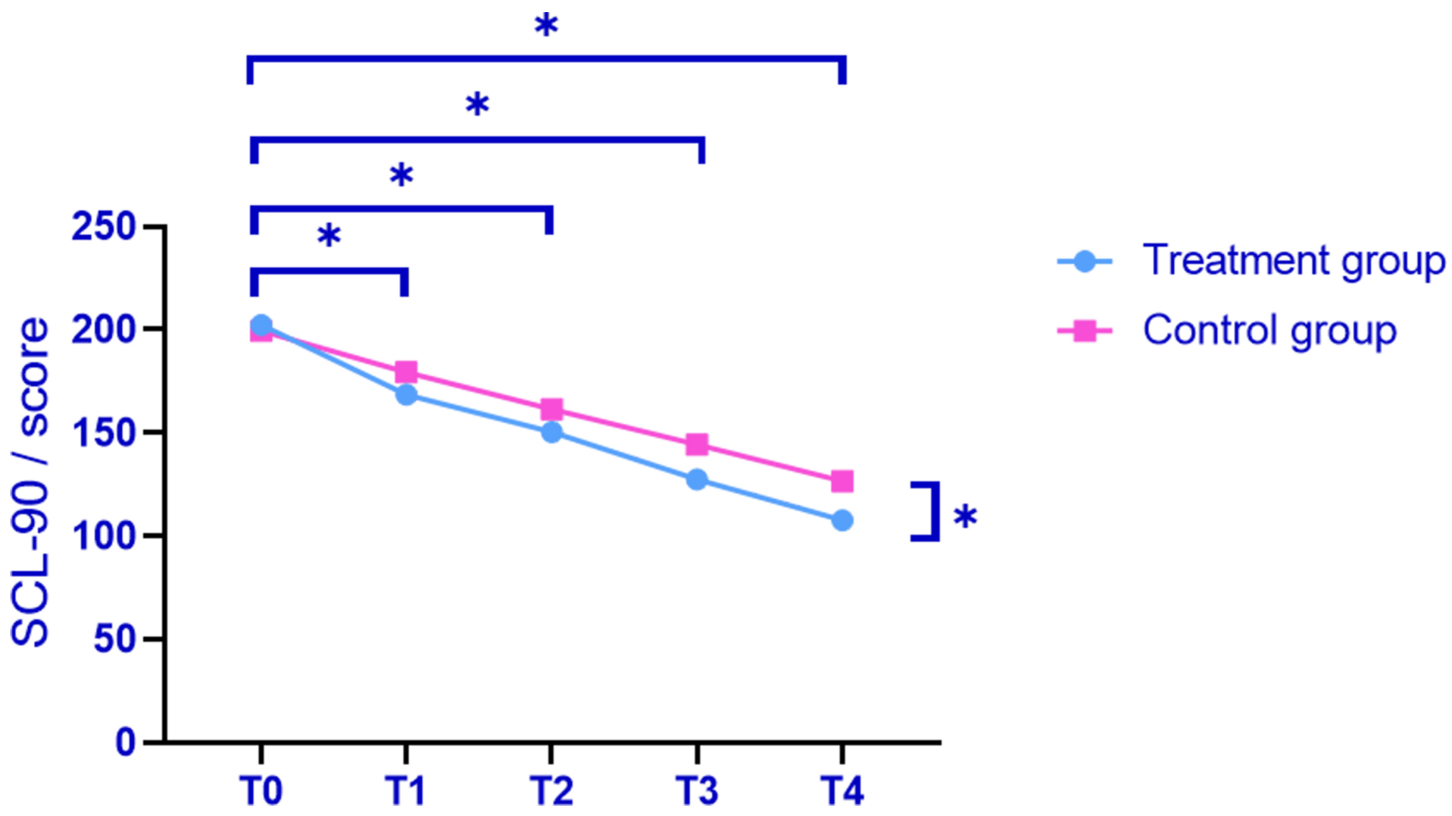
**Pre- and post-treatment SCL-90 score comparison**. Note: *p < 0.05 (after Bonferroni correction); SCL-90, Symptom Checklist 90.

Relative to the baseline (T0), both groups exhibited a significant reduction in SCL-90 scores at T1, T2, T3 and T4 (*p* < 0.05). At T0, the SCL-90 scores were not significantly different between the two groups based on further analysis (*p* > 0.05). At T1, T2, T3 and T4, the treatment group demonstrated significantly lower SCL-90 scores compared to the control group (*p* < 0.05, after Bonferroni correction; Fig. 2). A repeated-measures ANOVA was conducted to analyse variations in SCL-90 scores over time between the two groups. The results of Mauchly’s sphericity test for SCL-90 scores indicated that the sphericity assumption was met (*p* > 0.05), thereby negating the necessity for adjustment using the Greenhouse-Geisser method. Significant effects were observed for group, measurement times, and their interaction concerning SCL-90 scores (F1 = 36.917, F2 = 253.552, F3 = 4.382, *p* < 0.05, after Bonferroni correction).

### Comparison of CGI-SI Scores

**Fig. 3.**
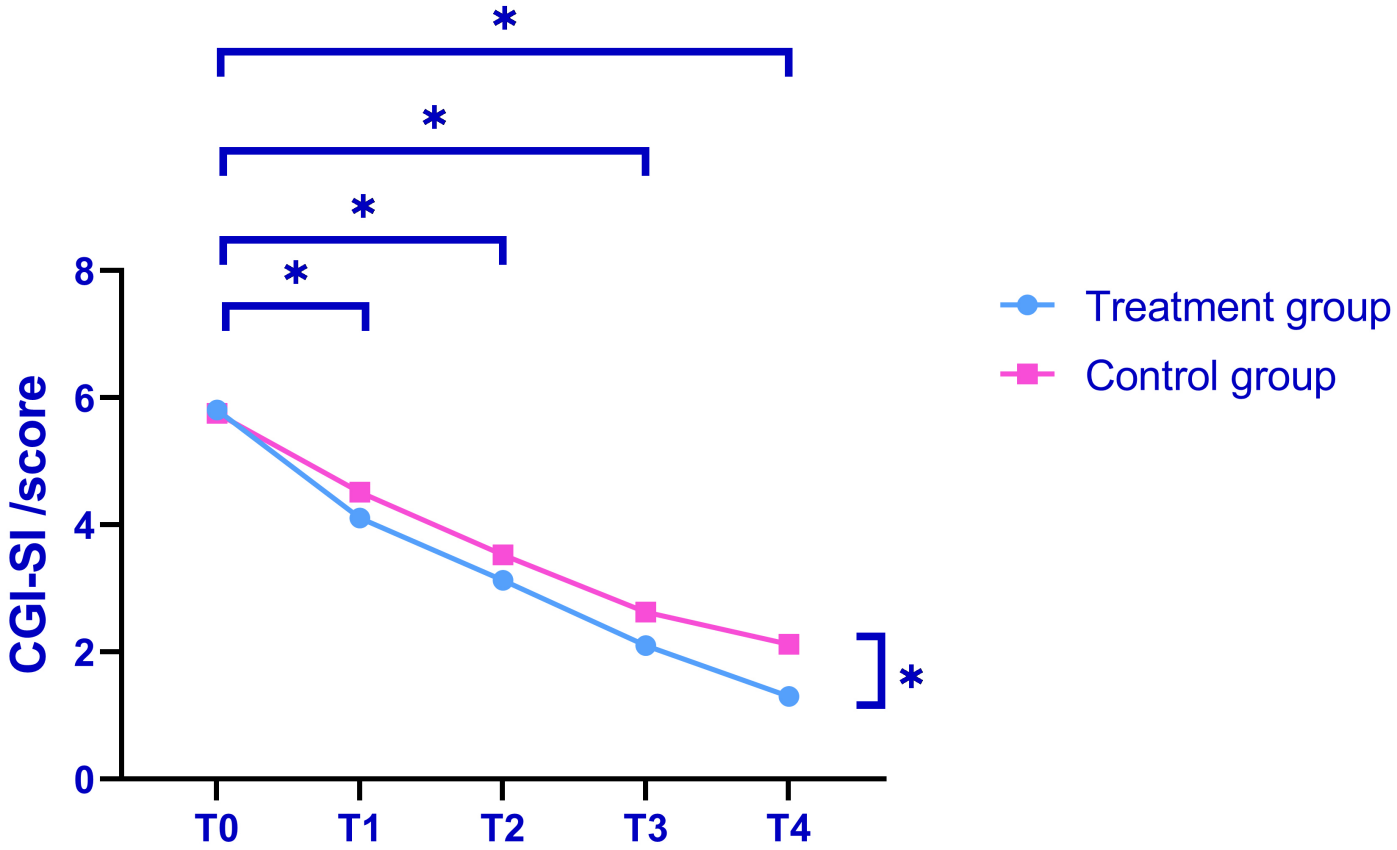
**Analysis of CGI-SI scores pre- and post-treatment**. Note: *p < 0.05 (after Bonferroni correction); CGI-SI, Clinical Global Impression-Severity of Illness.

During the pre-intervention phase, the CGI-SI scores were similar across groups (*p* > 0.05). Post-intervention, the treatment group exhibited significantly lower CGI scores compared to the control group (*p* < 0.05, after Bonferroni correction; Fig. 3). A repeated-measures ANOVA was utilised to evaluate the CGI-SI scores at various time points for both groups. The Mauchly’s sphericity test indicated a violation of the sphericity assumption for CGI-SI scores (*p* < 0.05). Following correction via the Greenhouse-Geisser method, the main effects of group, measurement times and their interaction were all found to be significant for CGI-SI scores (F1 = 56.900, F2 = 712.770, F3 = 5.467, *p* < 0.05, post-Bonferroni correction).

### Clinical Efficacy Assessment

The efficacy of treatment in the treatment group reached 88.89%, markedly surpassing the control group’s 68.25% (*p* < 0.05; Table 2).

**Table 2.** **Evaluation of treatment outcomes (n [%])**.

Group	Marked improvement	Improvement	Absence of improvement	Effective rate in total
Treatment group (n = 63)	32 (50.79)	24 (38.10)	7 (11.11)	56 (88.89)
Control group (n = 63)	11 (17.46)	32 (50.79)	20 (31.75)	43 (68.25)
*p*				0.005

### Comparison of TESS Scores

**Fig. 4.**
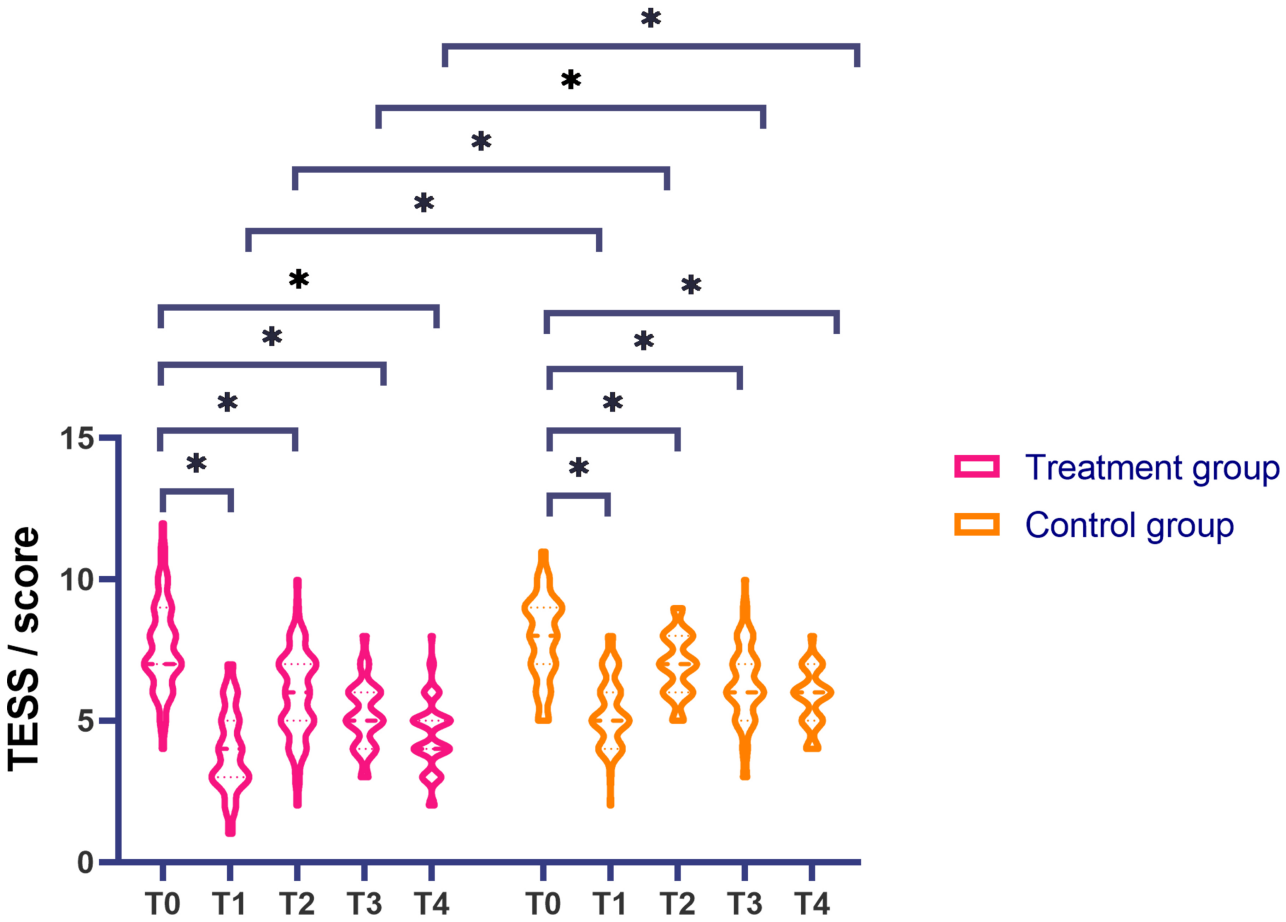
**Analysis of TESS scores pre-treatment and post-treatment**. Note: *p < 0.05 (after Bonferroni correction); TESS, Treatment Emergent Symptom Scale.

Analysis of TESS scores across various treatment stages for both groups revealed that at each measurement time point, the treatment group exhibited significantly lower scores compared to the control group (*p* < 0.05, after Bonferroni correction; Fig. 4). Repeated-measures ANOVA was utilised; the results of Mauchly’s sphericity test for TESS scores indicated that the sphericity assumption was satisfied (*p* < 0.05). Following correction via the Greenhouse–Geisser method, the main effects of group and measurement times, along with their interaction, were found to be significant for TESS scores (F1 = 102.991, F2 = 98.896, F3 = 3.420, *p* < 0.05, after Bonferroni correction).

### Adverse Reactions

The treatment group exhibited a lower incidence of adverse reactions compared to the control group (*p* < 0.05), with the majority being mild, including somnolence, dizziness, nausea, dry mouth, constipation and anxiety. Patients exhibited good tolerability, with no serious adverse events reported, indicating the safety of the combined treatment (Table 3).

**Table 3.** **Adverse reactions (n [%])**.

Group	Somnolence	Dizziness	Nausea	Dry mouth	Constipation	Anxiety	Total incidence
Treatment group (n = 63)	1 (1.58)	2 (3.17)	3 (4.76)	4 (6.35)	2 (3.17)	0 (0.00)	12 (19.05)
Control group (n = 63)	4 (6.35)	5 (7.94)	4 (6.35)	5 (7.94)	0 (0.00)	4 (6.35)	23 (36.51)
χ ^2^							4.787
*p*							0.029

### Single Factor Analysis of Clinical Efficacy 

The univariate analysis indicated that the ineffective group had a greater proportion of patients on venlafaxine monotherapy, an older average age and lower baseline HAMA scores (HAMA-T0) compared to the effective group (*p* < 0.05; Table 4).

**Table 4.** **Single-factor analysis of clinical efficacy**.

Factor		Ineffective group (n = 27)	Effective group (n = 99)	t/χ^2^	*p*
Age ($\bar{x}$ ± s, years)		35.41 ± 11.23	30.83 ± 9.65	2.109	0.037
Sexuality (n [%])	Male	11 (40.74)	42 (42.42)	0.025	0.875
	Female	16 (59.26)	57 (57.58)		
Course of disease ($\bar{x}$ ± s, years)		4.27 ± 1.33	4.47 ± 1.57	0.582	0.561
Marriage (n [%])	Wedlock	8 (29.63)	25 (25.26)	0.575	0.750
	Non-married	11 (40.74)	37 (37.37)		
	Divorced/widowed	8 (29.63)	37 (37.37)		
Treatment (n [%])	Venlafaxine treatment	20 (74.07)	43 (43.43)	7.966	0.005
	Venlafaxine combined with rTMS treatment	7 (25.93)	56 (56.57)		
HAMA/score	T0 (M [Q1, Q3])	26.00 (24.00, 28.00)	27.00 (25.00, 29.00)	6.749	0.009
	T1 (M [Q1, Q3])	24.00 (22.00, 26.00)	23.00 (21.00, 25.00)	3.476	0.062
	T2 ($\bar{x}$ ± s)	18.04 ± 4.94	18.40 ± 4.73	0.354	0.724
	T3 ($\bar{x}$ ± s)	13.48 ± 4.07	11.89 ± 3.74	1.923	0.075
	T4 (M [Q1, Q3])	9.00 (7.00, 11.00)	8.00 (7.00, 10.00)	1.852	0.173
HAMD/score	T0 ($\bar{x}$ ± s)	25.56 ± 2.65	25.89 ± 2.60	0.588	0.557
	T1 ($\bar{x}$ ± s)	22.19 ± 4.78	21.77 ± 3.74	0.483	0.630
	T2 ($\bar{x}$ ± s)	16.67 ± 3.03	15.88 ± 2.96	1.220	0.225
	T3 ($\bar{x}$ ± s)	13.19 ± 3.73	12.13 ± 3.93	0.747	0.456
	T4 (M [Q1, Q3])	9.00 (7.00, 11.00)	9.00 (7.00, 12.00)	0.316	0.574
CGI-SI/score (M [Q1, Q3])	T0	6.00 (5.00, 6.00)	6.00 (5.00, 6.00)	0.235	0.628
	T1	4.00 (4.00, 5.00)	4.00 (4.00, 5.00)	0.102	0.749
	T2	3.00 (3.00, 4.00)	3.00 (3.00, 4.00)	0.602	0.438
	T3	3.00 (2.00, 3.00)	2.00 (2.00, 3.00)	1.651	0.199
	T4	2.00 (1.50, 2.00)	2.00 (1.00, 2.00)	0.542	0.462
TESS/score (M [Q1, Q3])	T0	8.00 (7.00, 9.00)	8.00 (7.00, 9.00)	1.233	0.269
	T1	5.00 (4.00, 6.00)	5.00 (3.00, 6.00)	1.236	0.266
	T2	7.00 (6.00, 8.00)	7.00 (5.00, 7.00)	2.215	0.137
	T3	6.00 (5.00, 7.00)	6.00 (5.00, 6.00)	0.502	0.479
	T4	5.00 (5.00, 6.00)	5.00 (4.00, 6.00)	0.860	0.354

Notes: T0: Baseline; T1, T2, T3, T4: 1, 2, 4 and 6 weeks after treatment initiation, respectively. HAMA, Hamilton Anxiety Rating Scale; HAMD, Hamilton Depression Rating Scale; CGI-SI, Clinical Global Impression-Severity of Illness; TESS, Treatment Emergent Symptom Scale; rTMS, repetitive transcranial magnetic stimulation.

### Multivariate Analysis 

The clinical efficacy of the patient was assessed as the dependent variable, categorised as effective (0) or ineffective (1). The independent variables included the treatment method (venlafaxine treatment = 1, venlafaxine combined with rTMS treatment = 0), HAMA (T0; original value input) and age (original value input). Multivariate logistic regression analysis indicated that venlafaxine treatment (odds ratio [OR] = 3.181, 95% confidence intervals [CI] [1.184–8.549]) and HAMA (T0) level (OR = 0.784, 95% CI [0.644–0.954]) were significant factors influencing the clinical efficacy in patients (*p* < 0.05; Table 5).

**Table 5.** **Multi-factor analysis**.

Factor	B	SE	Wald/χ^2^	*p*	OR	95% CI
Treatment/venlafaxine treatment	1.157	0. 504	5.265	0.022	3.181	1.184–8.549
HAMA/T0	–0.243	0.100	5.881	0.015	0.784	0.644–0.954
Constant	2.879	2.723	1.118	0.290	17.803	

Note: Adjust age as a covariate. HAMA, Hamilton Anxiety Rating Scale.

## Discussion

SD is a mental disorder characterised by the presence of somatic symptoms that frequently lead to anxiety and depression in patients. Additionally, some individuals may experience sleep disorders and cognitive decline [15, 16]. Patients exhibit a pronounced preoccupation with or concern regarding their somatic symptoms [17, 18]. Recent years have seen an increase in the incidence of SD, attributed to the combined effects of considerable life burdens and elevated psychological stress. This trend adversely affects the physical and mental health of patients but imposes substantial burdens on families and society [17]. Therefore, exploring effective and safe treatment protocols is a key focus of clinical research. Venlafaxine effectively and rapidly alleviates anxiety and depressive symptoms, demonstrating favourable therapeutic outcomes, minimal side effects and a high safety profile [19, 20, 21]. Venlafaxine inhibits the reuptake of 5-HT and NE at the synaptic cleft, with additional inhibitory effects on dopamine reuptake, thereby alleviating anxiety, depression and somatic discomfort in patients [22, 23]. rTMS is a novel non-invasive and painless therapeutic approach that targets specific regions of the cerebral cortex using magnetic signals. This method depolarises neuronal cells, induces the generation of electrical currents, enhances neuronal activity and ultimately fulfills therapeutic objectives [7]. In this study, the application of rTMS in conjunction with venlafaxine for the treatment of SD resulted in a total effective rate of 88.89% in the intervention group, which was significantly higher than the 68.25% observed in the group receiving venlafaxine alone (*p* < 0.05). The findings suggest that the combination of rTMS and venlafaxine is more effective for treating SD compared to venlafaxine alone.

This study found that the HAMD, HAMA and SCL-90 scores of both groups significantly decreased post-treatment, indicating that venlafaxine effectively improves depressive, anxious and somatic symptoms, demonstrating its efficacy in treating somatoform disorders. The analysis indicates that the combined treatment protocol utilises venlafaxine to inhibit 5-HT reuptake, which enhances the activity and duration of various neurotransmitters, leading to a sustained increase in neuronal excitability. The adjunctive use of rTMS can quickly modulate nerve cell action potentials in the short term, facilitating the activation of excitability in specific cortical areas of the brain, thereby allowing them to autonomously engage in emotional regulation. Magnetic stimulation facilitates positive alterations in cerebral blood flow and neural tissue, contributing to the repair of the nervous system and enhancement of cognitive and neural functions, aligning with findings from prior studies [23, 24]. These consistent findings offer significant insights for future clinical practice. Some studies suggest that for patients experiencing dominant somatic discomfort, monotherapy with antidepressants is ineffective [25, 26], and adverse drug reactions frequently result in treatment discontinuation. Therefore, patients are more likely to accept non-pharmacological treatments.

The pathogenesis of somatoform disorders may be associated with alterations in the body’s 5-HT levels and its receptors [27]. rTMS is a painless and non-invasive therapeutic approach that has demonstrated improvements in cognitive functions [28, 29] and enhanced therapeutic efficacy, addressing the limitations of pharmacotherapy. rTMS can influence local cortical function by adjusting its stimulation frequency to induce either excitation or inhibition. Clinical studies [9, 30] have demonstrated that low-frequency rTMS reduces neuronal activity levels, whereas high-frequency rTMS increases them. The mechanism of action of rTMS directly influences prefrontal cortical function and indirectly affects subcortical structures within anxiety-related neural circuits, thereby balancing emotional regulation within the circuit. This method aids in regulating abnormal neural circuit activity linked to specific psychiatric disorders [31, 32]. Recent studies have confirmed that rTMS can modulate 5-HT levels [33, 34], indicating that this intervention may serve as a foundational approach for treating somatoform disorders. This study utilised a combination of venlafaxine and rTMS for the treatment of somatoform disorders. Comparison of the HAMA and HAMD scores between the two groups at T0, T1, T2, T3 and T4 indicated a decline in scores for both groups after 6 weeks of treatment. The treatment group demonstrated a more significant reduction compared to the comparison group. The mechanism suggests that rTMS enhances cortical excitability and restores functional asymmetry between the left and right hemispheres of the brain, leading to improvements in depression, anxiety and chronic pain [35, 36].

The study indicated that the treatment group showed a significant reduction in SCL-90 scores from T0, with statistically significant differences noted when compared to the control group. This can be ascribed to the synergistic effectiveness of rTMS and venlafaxine in rapidly alleviating symptoms. Venlafaxine demonstrates fundamental anti-anxiety and antidepressant properties through the rapid elevation of 5-HT and NE levels. Additionally, rTMS has the capacity to inhibit hyperactive neural circuits within the prefrontal cortex. The integration of the two can effectively alleviate symptoms of anxiety and depression by regulating emotional homeostasis. Furthermore, rTMS directly modulates brain regions associated with somatosensory perception, including the insula and anterior cingulate cortex [30, 37]. This addresses abnormal processing of somatosensory signals and, along with the indirect decrease in sensitivity to somatic symptoms resulting from enhanced mood, provides significant relief from somatic discomfort via two mechanisms: neural circuit regulation and symptom perception [35]. Moreover, the present study assessed adverse reactions to pharmacotherapy in both groups. The findings demonstrated that the adverse reactions were mild and manageable, with spontaneous relief occurring as patients acclimated to the medications. Venlafaxine exhibits minimal to negligible affinity for muscarinic cholinergic, histamine H1 or adrenergic α1 receptors, thereby enhancing its favorable side-effect profile and overall safety [38, 39]. Post-treatment, the TESS scores for the treatment group at each time point were lower than those of the control group, suggesting that rTMS combined with venlafaxine demonstrated superior safety and tolerance in addressing SD. The TESS scores for both groups at T2 were marginally increased relative to those at T1 likely because T2 was the early stage of treatment, where the dosage of venlafaxine was increased from 75 mg/day to 150 mg/day within the first week of treatment. Patients may not completely acclimate to the increased dosage at T2, leading to a mild exacerbation of drug-related adverse effects, including nausea and drowsiness. Patients’ awareness of physical discomfort remains significant, leading to an increased likelihood of subjective reporting of adverse reactions. The total incidence of adverse reactions in the treatment group was considerably reduced likely because of the synergistic effect of rTMS. This intervention effectively alleviates anxiety symptoms and aids patients in diminishing their focus on physical symptoms, thereby rapidly decreasing physical discomfort.

This study categorised patients into ineffective and effective groups based on post-treatment outcomes to investigate the specific factors influencing clinical efficacy. Univariate analysis revealed that the ineffective group had a greater proportion of patients on venlafaxine monotherapy, were older and presented with lower baseline HAMA scores (HAMA-T0) than the effective group (*p* < 0.05). Multivariate logistic regression analysis identified venlafaxine monotherapy and baseline HAMA score (HAMA-T0) as significant factors affecting clinical efficacy (*p* < 0.05). The combination of venlafaxine and rTMS demonstrates higher efficacy in alleviating symptoms in patients with SD than venlafaxine administered alone. The combined treatment likely enhances the efficacy of venlafaxine through rTMS neuromodulation and mitigates the side effects associated with monotherapy. For patients exhibiting mild anxiety symptoms, the combined treatment may effectively augment the antidepressant and anti-anxiety effects through synergistic mechanisms. The non-invasive neuromodulation of rTMS may facilitate the recovery of brain function, thereby enhancing the overall treatment efficacy. This study demonstrates the potential therapeutic effects of venlafaxine combined with 15 Hz rTMS for SD. However, its findings are constrained by a small sample size and the lack of placebo control groups (venlafaxine + placebo and sham rTMS + venlafaxine). This complicates the ability to accurately differentiate the genuine therapeutic effects of rTMS from placebo effects. The absence of comparisons with varying rTMS frequencies and sham stimulation may compromise the validity of the results. Future research will utilise a double-blind randomised controlled design to compare true and sham rTMS, increase the sample size and prolong the follow-up period to strengthen the evidence base.

## Conclusion

This study compared the efficacy and safety of rTMS combined with venlafaxine against venlafaxine alone in the treatment of SD. The combined therapy was more effective and safe than monotherapy and thus recommended for clinical application. Age, treatment modality and baseline HAMA (T0) were found to be critical factors affecting efficacy. Clinicians applying this combined regimen must consider patient age and baseline anxiety level (HAMA-T0) when evaluating treatment response. This approach emphasises the importance of efficacy monitoring and individualised adjustments for older patients or those with elevated anxiety levels to improve therapeutic outcomes and patient adherence.

## Availability of Data and Materials

All experimental data included in this study can be obtained by contacting the corresponding author if needed.
